# Peripheral Circulating Blood Cells Deviation Based on Tumor Inflammatory Microenvironment Activity in Resected Upstaged Lung Adenocarcinomas

**DOI:** 10.3390/jcm13247597

**Published:** 2024-12-13

**Authors:** Alessandro Bonis, Francesca Lunardi, Giulia Pagliarini, Vincenzo Verzeletti, Luigi Lione, Alberto Busetto, Giorgio Cannone, Giovanni Maria Comacchio, Marco Mammana, Eleonora Faccioli, Alessandro Rebusso, Marco Schiavon, Samuele Nicotra, Andrea Dell’Amore, Federico Rea

**Affiliations:** 1 Thoracic Surgery Unit, Department of Cardiac, Thoracic, Vascular Sciences and Public Health—DSCTV, University of Padova, 35128 Padova, Italy; 2 Pathology Unit, Department of Cardiac, Thoracic, Vascular Sciences and Public Health—DSCTV, University of Padova, 35128 Padova, Italy

**Keywords:** tumor microenvironment, PD-L1, TILs, blood cells, lung adenocarcinoma, neutrophil-to-lymphocyte ratio

## Abstract

**Background**: The tumour inflammatory microenvironment (TIME) reflects a selective activation of the central immune system (IS), particularly T-cells expansion, which leads to immune cells migrating to the target, such as lung cancer, via the bloodstream and lymphatic vessels. In this study, the aim is to investigate whether the distribution of peripheral blood cells varies based on the immune status of patients with lung adenocarcinoma. **Methods**: This is a single-center retrospective study conducted in the Thoracic Surgery Unit of the University of Padua (Italy) between 1 January 2016 and 1 April 2024. It included patients (>18 years old) with lung adenocarcinoma deemed resectable (cT2bN0M0 or lower) who experienced pathological upstaging (IIB or higher). Patients were classified as TIME-active (with tumour-infiltrating lymphocytes—TILs and/or PD-L1 expression) or TIME-silent (without TILs or PD-L1). According to the TIME status, peripheral blood cell counts with clinical and pathological data were compared between groups using the Fisher’s, Pearson’s or Wilcoxon’s test when appropriate. A Kaplan–Meier estimator investigated overall survival (OS) and recurrence-free survival (RFS) adopting the log-rank test. **Results**: Preoperatively, the TIME-a group demonstrated a significantly higher lymphocyte count (*p* = 0.02) and a lower absolute neutrophil rate (*p* = 0.01) than TIME-s. These differences persisted after resection (*p* = 0.06 and *p* = 0.02) while they became similar one month after surgery (*p* = 1 and *p* = 0.32). The neutrophil-to-lymphocyte ratio—NLR showed similar trends (*p* = 0.01 and *p* = 1). Better OS and RFS were shown in the TIME-a group (*p* = 0.02 and 0.03, respectively). **Conclusions**: Resected upstaged lung adenocarcinomas show distinct peripheral blood cell profiles based on immune status. TIME-active patients had a significantly lower NLR, which normalized post-surgery. Surgical resection may help restore native immune surveillance.

## 1. Introduction

Lung cancer (LC) remains one of the leading causes of death worldwide and the International Agency for Research on Cancer continues to release alarming reports on its epidemiology across developed and emerging countries. As a silent killer, LC is generally diagnosed in an advanced stage and surgically upfront resectable cases are limited [1]. Consequently, cancer research has developed several different fields in order to understand the deepest mechanisms regulating cancer initiation and progression.

The immune system (IS) has emerged as one of these novel research fields, drawing attention in recent years for its favorable prognostic and predictive roles across various tumors [2]; in lung cancer, this has led to the introduction of several innovations in daily practice, including adjuvant and neoadjuvant treatments [3,4,5,6], with the most notable advancements stemming from new insights into cell-to-cell interactions, both among immune cells and in the context of tumor-to-immune cell crosstalk [7,8,9]. Tan et al. clearly described the deep relationship between lung cancer and inflammation, highlighting that immune cells are responsible for several processes that modulate cancer progression.

This background has consolidated the idea of a tumor immune associated microenvironment (TIME) in which several chemotactic molecules and signals produce a multifaceted response against cancer, thus resulting in the immune control of the tumor and acting as a barrier to its progression [10]. The TIME is fairly known and diffusely investigated, while the evidence of blood peripheral elements reflecting an active immune peritumoral tissue is not well established. Nevertheless, as previously described by Lu Y. et al., investigating changes in circulating peripheral immune blood cells driven by the active cancer-associated microenvironment found that the neutrophil-to-lymphocyte ratio (NLR) is usually altered, suggesting that NLR may serve as biomarker [11,12]. These studies were primarily designed to predict the response to immune checkpoint inhibitors, but broader meanings may be hidden in the peripheral white blood cell variation [13,14]. For instance, the hypothesis of a blood discrepancy between immune-active cancers and immune-silent lung tumors has been reported in preclinical studies, and different immunotypes have been described and investigated regardless of response to treatments [15,16]. In fact, the central priming after a cancer antigen presentation process produces as a result a selective immune activation and expansion against tumor with a blood or lymphatic peripheral migration of the active lymphocytes to the target (antigen-expressing cancer cells) and a selective killing process due to tumor infiltrating lymphocytes (TILs) [17,18]. Consequently, this elimination phase induces a cancer immunoediting step with the expression of the programmed death ligand (PD-L1) that is prone to hide the tumor against TILs [19]. Considering this delicate equilibrium, it is plausible that circulating immune cells may exhibit a blood peripheral variation following cancer priming not only in an advanced setting, as previously reported [20], but also in upstaged locally advanced or in early-staged relapsed scenarios, in which a curative intent, in selected cases, is still an achievable goal.

This study aims to verify whether peripheral blood circulating cells show any absolute quantitative differences between patients with an active tumor inflammatory microenvironment (TIME), marked by PD-L1 expression and/or the presence of tumor-infiltrating lymphocytes (TILs), and those with a silent immune profile, acting as a biochemical marker of an activated adaptive immune surveillance. As a secondary aim, this study was designed to verify any overall survival and recurrence-free survival difference according to the TIME status.

## 2. Materials and Methods

This is a retrospective monocentric study that retrieved data of patients with early-stage lung adenocarcinoma (ADC) who underwent surgical treatment (lobectomy or segmentectomy) at University Hospital, Thoracic Surgery Unit of Padova (Italy) between 1 January 2016 and 1 April 2024. This model was previously proposed to investigate the role of the tumor inflammatory microenvironment in unexpected, escaped lung tumors [21].

This study was conceptualized in line with the Declaration of Helsinki, and all patients gave informed consent to participate in the research activity of the department. No specifical Ethical Committee approval was required as blood examinations used are routinely performed during the daily clinical practice.

### 2.1. Population, Inclusion and Exclusion Criteria

The study included patients with primary ADC (≥18 years old), managed with anatomical resection via lobectomy or segmentectomy and simultaneous radical hilar and mediastinal lymphadenectomy for early-stage tumors, ranging from cT1a1N0M0 to cT2bN0M0, in accordance with the 8th Edition of the TNM classification, and who had an upstaged pathological diagnosis for lymph nodal involvement, pleural involvement or T3 or T4 primary tumor [21].

The evaluation of the clinical stage was made with an enhanced total-body CT scan and 18-FDG PET/CT. According to previous studies [22,23], lymph nodes were defined as negative if the diameter was less than one centimeter on a chest CT-scan and with a SUVmax (Positron Emission Tomography Maximum Standard Uptake Value) less than five. All the radiological investigations had to be performed at most one month before surgery to be acceptable.

Data were collected from patients’ past medical history and preoperative examinations. The variables evaluated were age, sex, Body Mass Index (BMI), the smoking habitude, alcohol abuse, diabetes, hypertension and the Charlson Comorbidity Index (CCI). A preoperative pulmonary function test was routinely ruled out registering the Forced Vital Capacity (FVC), the Forced Expiratory Volume in one second (FEV1) and the alveolar carbon monoxide diffusion limit (DLCO/VA). Moreover, among the preoperative variables, we also collected clinical staging (cTNM) data such as tumor maximum diameter and nodal status. The intraoperative variables include surgical time, surgical access type, conversion to open surgery, presence of pleural adherence, bleedings and number of lymph nodes resected, which were collected from the patient’s medical record, as well as the main postoperative surveillance data.

The authors excluded any type of non-anatomical lung resection and patients who underwent neoadjuvant therapies. Moreover, the following were excluded: (a) patients with a story of any type of blood cancer or blood-related peripheral cell disfunction; (b) patients undergoing treatments with steroids due to any type of disease (i.e., autoimmune, allergic, oncological, respiratory); (c) patients already surgically treated with wedge resections or judged suitable for a bilobectomy or pneumonectomy at preoperative evaluation.

### 2.2. Pathological and Biochemical Examination

All pathological data were collected analyzing resected specimen such as histotype and other histological characteristics of the lesion like growth pattern, grading, number of mitoses, necrosis, lymphocytic infiltrate (TILs), STAS (Spread Through Air Spaces), fibrosis, vascular invasion, pleural invasion, extent of resection margins, number of harvested lymph nodes and pathological TNM staging. The expression of PD-L1 was also collected as a percentage and evaluated as a tumor proportion score (TPS).

The presence of TILs (at least 10%) and/or PD-L1 (at least 1%) expression were the inclusion criteria for the TIME-a group (Tumor Inflammatory Microenvironment—active) while the absence of tumor infiltrating lymphocytes and programmed-death ligand-1 expression was necessary to consider the patient as TIME-s (Tumor Inflammatory Microenvironment—silent).

Blood exams have routinely performed at preoperative evaluation, which occurred roughly 30 days before surgery, on the first post-operative day, and at the first oncological visit (nearly 30 days after surgery). We collected data about platelets (plt), white blood cell (WBC) differential count, CRP (C-Reactive Protein) and monocytes. The white blood cell differential count was evaluated by percentage due to the different measuring machines in the considered timeline. The neutrophil-to-lymphocyte ratio was obtained dividing the absolute percentage of both variables.

### 2.3. Statistical Analysis

Our tables report continuous variables as medians and interquartile ranges (IQRs) while categorical ones are presented as absolute numbers and frequencies. TIME-a and TIME-s groups were compared using the Fisher’s exact test, Pearson’s Chi-squared test or Wilcoxon’s test when appropriate. Biochemical differences were graphically reported as a box and whiskers plot.

Kaplan–Meier estimator was used for overall survival (OS) and for relapse (Recurrence-Free Survival—RFS) and curves were compared adopting the log-rank test. Survival time was calculated from the surgical date to death. RFS was obtained as the time between surgery and the first radiological evidence of any type of recurrence (local, regional or distant), confirmed after a Tumor Board discussion. Univariate and Multivariate Cox Regression analyses for survival were provided in comparison with pathological variables.

Analyses and plots were performed using Jamovi software (v2.3.21) and R statistical software (v4.2.2-R Core Team 2022) using the survival package. Significance was set at *p* ≤ 0.05.

## 3. Results

### 3.1. Population and Pathological Data

This study included 61 patients in the considered timeline with a median age of 71 years old (IQR 63–75), a normal Body Mass Index (24 kg/m^2^) and a slight prevalence of female gender (59%) The Charlson Comorbidity Index indicated a median score of 4 points. Thirty-three patients (54%) were active smokers at the diagnosis. Concerning preoperative respiratory performance, the median values of respiratory volumes and alveolar diffusion were permissive to lobectomy and any of the segmentectomy performed was related to marginal lung function. Surgery was performed through a minimally invasive approach (VATS—Video Assisted Thoracic Surgery in 98% of procedures while 2% were RATS—Robot Assisted Thoracic Surgery). In two cases (3.3%), it was necessary to convert in thoracotomy the intervention due to a minor bleeding (one case of pulmonary artery small branch rupture) and one case of adherences that did not allow the hilar structures to be safely isolated. The median hospital length was 4 days (IQR 3–6) (Table 1).

As concerns perioperative data, a median diameter at a preoperative CT-scan of 25 mm lung nodule underwent surgery with a predominant cT1cN0M0 stage (24 cases—39%). The median maximum Standard Uptake Value at preoperative positron emission tomography was almost 8 (IQR 5.3–10.3). The pathological report showed a predominant pT2a stage (26 cases, 43%), a slight predominance of IIB (33 cases, 54%) and a median resection margin of 20 mm. A spread through air space was represented in almost half of patients (29 cases, 48%). An acinar histotype was generally discovered (36 cases, 59%) and the solid one followed with 14 cases (23%). The pathological grading was comparable (almost half of G2 and a half of G3), showing a general scarce or moderate differentiation of adenocarcinomas. A total of 35 cases (57%) had a peritumoral infiltration of lymphocytes with 32 cases (52%) of vascular invasion and 42 specimens with at least microscopic visceral pleural involvement (70%). A total of 29 specimens (almost 50%) showed a PD-L1 TPS ≥ 1%. In total, a tumor microenvironment was found to be activated in 46 cases (75% of the sample). Further details are reported in Table 2. When comparing the radiological preoperative size, the Maximum Standard Uptake Value at the Positron Emission Tomography and pathological variables of TIME-a and TIME-s cohorts, any of them, with the exception of the Grading (*p* = 0.05), were significantly different. In particular, G3 stains were 20% more present in TIME-a patients while TIME-s presented a higher quote of G1 or G2 ADCs (Table 3).

### 3.2. Circulating Blood Cells Analysis

**Figure 1:**
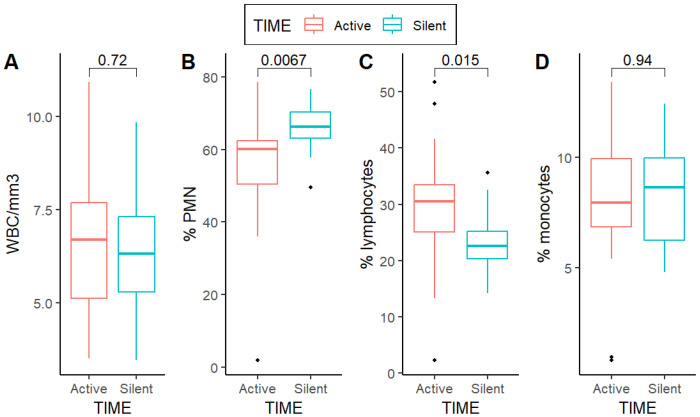
Preoperative values of peripheral circulating white blood cells. (**A**) WBC comparison between TIME-a and TIME-s; (**B**) PMN comparison between TIME-a and TIME-s; (**C**) Lymphocytes comparison between TIME-a and TIME-s; (**D**) Monocytes comparison between TIME-a and TIME-s.

Concerning peripheral blood examinations (Table 4), a higher preoperative blood lymphocyte rate in TIME-a patients (*p* = 0.02) and a lower neutrophil (PMN—polymorphonucleates) rate (*p* = 0.01) were observed (Figure 1).

**Figure 2:**
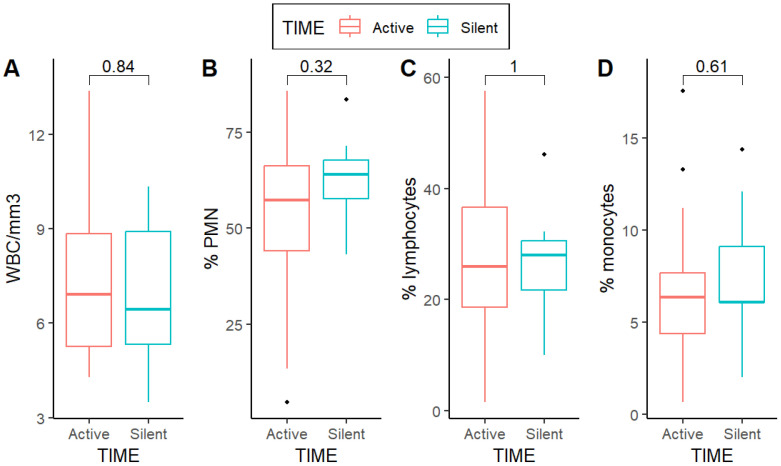
Postoperative values of peripheral circulating white blood cells. Differences seemed to be leveled one month after surgery, at the oncological evaluation before starting any adjuvant therapy. (**A**) WBC comparison between TIME-a and TIME-s; (**B**) PMN comparison between TIME-a and TIME-s; (**C**) Lymphocytes comparison between TIME-a and TIME-s; (**D**) Monocytes comparison between TIME-a and TIME-s.

Platelets were initially slightly higher than 10 percentage points in TIME-a patients (*p* = 0.1). In the first post-operative day, lymphocytes dramatically decreased, being only tendentially higher in the TIME-a group (*p* = 0.06). Circulating neutrophils increased in both groups, remaining significantly higher in TIME-s patients (*p* = 0.02) in the first postoperative day (Supplemental Material—Figure S1). At the oncological evaluation, almost one month after-surgery, this imbalance disappeared among groups being comparable to both neutrophils and lymphocytes (*p* = 0.32 and *p* = 1, respectively) (Figure 2).

**Figure 3:**
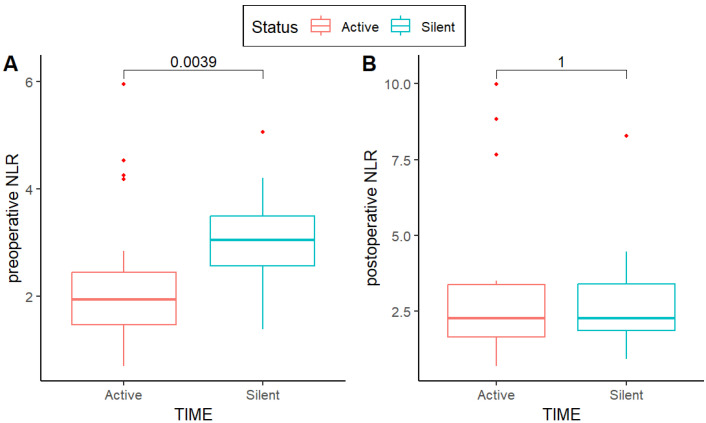
Neutrophil-to-lymphocyte ratio according to the immune status of patients, divided into preoperative (**A**) and postoperative (**B**) ratio.

As concerning the neutrophil-to-lymphocyte ratio, it was significantly different at preoperative evaluation (*p* < 0.01) while it was comparable one month after surgery at the oncological evaluation (*p* = 1) (Figure 3). Peripheral blood imbalances were significantly different while pathological variables among TIME-a and TIME-s patients remained comparable. Only the grading seemed to be remarkably higher in TIME-a patients (*p* = 0.05) but the authors cannot exclude the possibility that this may be related to the small sample size (Table 3). Due to the smoothing of values at oncological evaluation, we further assess whether peripheral blood circulating cell count may be linearly related to survival in months (OS) but none of these appeared to be significant. A slight unspecific tendency to significance was discovered in neutrophil rates (*p* = 0.1) that were positively related with OS (Supplemental Material—Figure S2).

**Figure 4:**
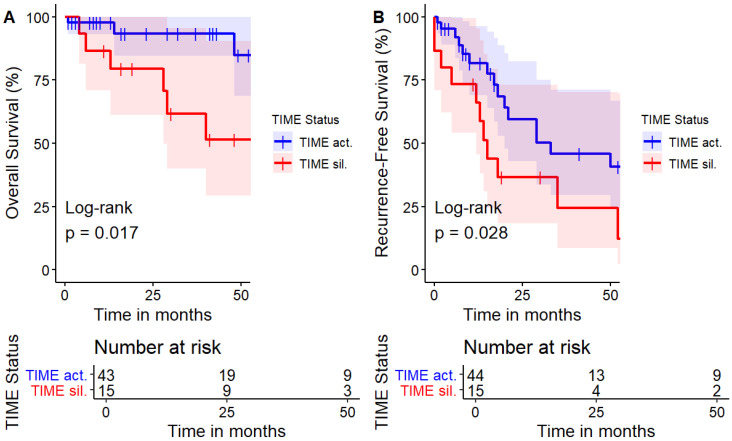
(**A**) Overall survival between the two groups; (**B**) recurrence-free survival comparison.

As concerns survival, according to the follow-up available, TIME-a patients showed a higher survival compared to the TIME-s cohort and this significance remained also in terms of recurrence-free survival according to the Log-rank test (*p* = 0.02 and *p* = 0.03—Figure 4). This independent effect on survival seemed to be confirmed by univariate and multivariate analysis for survival when considering other pathological variables available in the pathological report (Table 5).

## 4. Discussion

This research study aimed to investigate whether peripheral white blood cell count may present a different distribution between TIME-a and TIME-s resected lung ADCs, resulting in them being upstaged at pathological examination. This is a model specifically designed to evaluate the peripheral immune response against an unexpected, escaped cancer in patients with no preoperative treatments as a bridge to a feasible surgery, which is generally deserved in IIIA or IIIB stages [24]. Moreover, the timeline embraced a period that ended just before the oncological cares (adjuvant treatments) that may influence peripheral blood cells percentages [12].

The immune system is widely recognized as a critical protective predictor in lung cancer, being crucial not only for improving survival outcomes but also for enhancing the effectiveness of adjuvant immunotherapy in treatable cases. Among its components, tumor-infiltrating lymphocytes (TILs) have been demonstrated to be a favourable prognostic factor, providing valuable insights into disease progression and outcomes. Moreover, both TILs and PD-L1 expression have been extensively studied and reported as significant biomarkers, particularly relevant in predicting the response to immune checkpoint inhibitors (ICIs), thereby highlighting their essential role in shaping therapeutic strategies and advancing personalized medicine approaches in lung cancer [25,26,27,28,29,30].

An important novelty is that the absence of the Ligand-1 is related to a dysfunction of the beta-catenin/wnt molecular pathway, which is responsible for a cold microenvironment and a low efficacy of ICIs [19], suggesting that a PD-L1 negative stain could be surrounded by a cold tumor microenvironment (lack of infiltrating lymphocytes), which is represented in our model by the TIME-s sample.

Several studies reported a different peripheral blood cell distribution in lung cancer, both considering different stages and expressed markers [31] or after ICIs therapy [14]. In general, investigations have been conducted using the neutrophil-to-lymphocytes ratio, which demonstrated to be an independent worse predictor for survival [32]. A higher percentage of neutrophil was significantly associated with a lower OS in a single cohort of NSCLC, assessing that no connections are reported between tumor-associated macrophages and peripheral neutrophil count [33]. This is important for making the case that the higher peripheral count of neutrophils in our cohort was unrelated to cancer-associated macrophages that were not investigated in our study even considering that peripheral neutrophils are responsible for the margination in the innate immune response against hosts.

Based on our results, the peripheral white blood cell count seems to be differently distributed in resected upstaged adenocarcinomas. The imbalance between innate and acquired immune responses at the baseline is an intriguing finding. Moreover, it seemed to emerge that a significantly higher rate of circulating lymphocytes (and a consequently lower neutrophil-to-lymphocyte ratio—NLR) is expected to be registered in TIME-a lung adenocarcinomas (Figure 3A; NLR *p* = 0.004) with a subsequent restoration of postoperative peripheral ratio after surgery (Figure 3B; NLR *p* = 1) (Table 3).

An important study described that NLR variation may be an indirect peripheral signal of a raising antitumor response of the IS when the patient is treated with immunotherapy. In particular, a decreased NLR was associated with a significant better overall and progression-free survival [14]. This is in line with our findings, suggesting that a lower NLR (and a consequently higher peripheral lymphocyte quote) may be related to a TIME-a status.

Neutrophils represent the dominant cell type of the IS in NSCLC, and they act as a tumor-suppressor in an early stage of cancer, while in advanced cases they are widely accepted as tumor-promoters in different cancers with direct or indirect mechanisms [34,35,36]. Moreover, a lower NLR was previously associated with an increased T-cell tumor infiltration rate with favorable outcomes [37]. This point is in line with our findings (Figure 4) and could be justified with an increased immune cell-mediated surveillance in those who had an immune priming after a cancer-related antigen presentation and an enhanced native immune response in those patients that did not express an activated immune mechanism after pathological examination.

This is an indirect confirmation supporting the hypothesis that the immune system is differently activated among different patients with the same pathological lung cancer histotype (ADC) and pathological stage (IIB to IIIB). Mitchell and Colleagues published that the preoperative circulating absolute neutrophil rate was independently linearly correlated with the tumor burden while circulating lymphocytes failed to be statistically related. Moreover, the overall circulating neutrophils were reduced after lung resection with a recognized protumorigenic activity and an impaired antitumoral function [38]. This reinforces the role of the immune system not only as a predictor of adjuvant immunotherapy, which has already been confirmed, but also as a baseline favorable prognostic factor. As confirmation, the Log-Rank test on Kaplan–Meier estimates for survival remained significantly different between TIME-a and TIME-s patients (*p* = 0.02). This survival advantage was registered regardless of pathological differences that seemed to be insignificant for the exception of the tumoral grading, which was higher in TIME-a. Despite this, the survival of the TIME-a group was remarkably improved. It is possible that this grading difference may be attributable to the selection bias and the limited sample size.

Regarding the predictive value of the postoperative peripheral blood cell count, linear regression did not show any significant result; however, a higher postoperative percentage of circulating neutrophil was associated with an increased median OS. As reported by Mitchell and Colleagues, in our case a series of lower total peripheral neutrophil counts were also found after surgery [38]. Consequently, considering that postoperative neutrophil levels between TIME-a and TIME-s patients were newly balanced, it might be supposed that a postoperative restitution of an innate immunity surveillance instead of a still active circulating lymphocytes may be considered as a favorable trend, despite this slight tendency being far from acceptable as a conclusion in our sample and wider experiences being expected to confirm or decline this suspicion. The final surrounding idea is that by removing the primary stimulus (resected lung cancer), we assist to a switch-off of the immune system against the specific antigen that induced T-cells expansion and a restored native surveillance. For sure, this trend of lymphocytes percentage variation during perioperative timeline is intriguing but it must be clear that a certain conclusion is not yet possible. It is possible that by studying the neutrophil-to-lymphocyte relation as a ratio, this trend of lymphocytes was hidden.

Finally, it remains to be discussed how the switch-off of the immune system after resection (if acceptable as a theory and further confirmed) could be related to a successful or ineffective postoperative immunotherapy. The paucity of data pertaining to postoperative adjuvant immunotherapy in the considered sample precludes any meaningful conclusions.

Giving our data almost a couple of relevant limitations needs to be underlined. First, it is not possible to assess the type of imbalance circulating lymphocytes discovered in peripheral blood samples and we did not separate them according to their antigen-related activation. Then, the small sample size remains a weak spot in this study. Nevertheless, the highly selected sample did not allow for a larger cohort to be found, and results should be addressed as preliminary data for further wider cohorts. Further studies are needed to explore the prognostic and therapeutic implications of these findings.

## 5. Conclusions

This study suggests that peripheral blood cell counts vary according to the immune status of the tumor microenvironment in resected upstaged lung ADC. TIME-a patients show higher preoperative lymphocyte counts and lower neutrophil rate compared to TIME-s patients, with these differences becoming comparable one month after surgery. These findings suggest a potential role for peripheral blood cells as biomarkers for immune activity in lung cancer and may be related to peripheral evidence of a restored immune surveillance after surgery.

## Figures and Tables

**Table 1:** Study population characteristics and perioperative data.

Characteristic	N = 61
Age at surgery (years)	71 (63, 75)
Gender	
Female	36 (59%)
Male	25 (41%)
BMI (Kg/m^2^)	24.0 (21.9, 27.1)
Diabetes	9 (15%)
Hypertension	24 (39%)
Smoking history	
no	26 (43%)
yes	33 (54%)
former	2 (3%)
FVC (%)	105 (96, 112)
FEV1 (%)	104 (95, 112)
DLCO/VA (%)	83 (62, 101)
Operatory time (mins)	110 (85, 135)
Conversion	2 (3.3%)
Harvested lymphnodes (n)	13 (10, 17)
N1 lymphnodes	5 (3, 8)
N2 lymphnodes	6 (3, 8)
Hospital lenght (days)	4 (3, 6)

Abbreviations: Data are represented as median (IQR); n (%). BMI: Body Mass Index; DLCO: Carbon Monoxide Alveolar Diffusion (percentage); FEV: Forced Expiratory Volume in 1 s (percentage); former smokers were identified as those patients that were at least six months of smoke-free; FVC: Forced Vital Capacity (percentage); N1: N1 stations; N2: N2 stations.

**Table 2:** Pathological characteristics of the sample.

Characteristic	N = 61
cT diameter (mm)	25 (20, 37)
cT	
cT1a	3 (4.9%)
cT1b	13 (21%)
cT1c	24 (39%)
cT2a	12 (20%)
cT2b	9 (15%)
Median Standard Uptake Value	7.9 (5.3, 10.3)
Preoperative Hystology	
No	27 (44%)
Yes	34 (56%)
pSTAGE	
IIB	33 (54%)
IIIA	25 (41%)
IIIB	3 (5%)
Alveolar diffusion (STAS)	
Absent	32 (52%)
Present	29 (48%)
Median resection margin (mm)	20 (10, 40)
Resection margin (cut-off 10 mm)	
<10	17 (30%)
>10	40 (70%)
Hystotype	
Lepidic	3 (4.9%)
Acinar	36 (59%)
Solid	14 (23%)
Papillar	5 (8.2%)
Mucinous	1 (1.6%)
Unreported	2 (3.3%)
Grading	
G1	2 (4%)
G2	29 (48%)
G3	29 (48%)
Necrosis	
Absent	37 (62%)
Present	23 (38%)
Tumor Infiltrating Lymphocytes	
<10%	26 (43%)
10–30%	24 (39%)
>30%	11 (18%)
TIME	
Active	46 (75%)
Silent	15 (25%)
Fibrosis	
Absent	28 (48%)
Present	30 (52%)
Vascular Invasion	
Absent	29 (48%)
Present	32 (52%)
Pleural Invasion	
Absent	18 (30%)
Present	42 (70%)

Data are presented as n (%) or median (IQR).

**Table 3:** Pathological comparison between TIME-a and TIME-s patients.

Characteristic	N	Active N = 46 (75%)	Silent N = 15 (25%)	*p*-Value
cT diameter (mm)	61	26 (21, 37)	23 (20, 38)	0.85
SUV T	54	8.5 (5.4, 10.1)	7.1 (4.8, 10.3)	0.86
Alveolar Diffusion	61			0.50
Absent		23 (50%)	9 (60%)	
Present		23 (50%)	6 (40%)	
Resection margin (mm)	57	23 (10, 40)	20 (10, 29)	0.39
Grading	60			0.05
G1		0 (0%)	2 (13%)	
G2		21 (47%)	8 (53%)	
G3		24 (53%)	5 (33%)	
Necrosis	60			0.88
Absent		28 (62%)	9 (60%)	
Present		17 (38%)	6 (40%)	
Fibrosis	58			0.45
Absent		20 (45%)	8 (57%)	
Present		24 (55%)	6 (43%)	
Vascular Invasion	61			0.20
Absent		24 (52%)	5 (33%)	
Present		22 (48%)	10 (67%)	
Pleural Invasion	60			>0.99
Absent		14 (31%)	4 (27%)	
Present		31 (69%)	11 (73%)	

SUV: Standard Uptake Value. Comparisons were investigated with Fisher’s, Wilcoxon’s and Pearson’s test when appropriate.

**Table 4:** TIME-a and TIME-s peripheral blood examination values.

	N	Active	Silent	*p*-Value
		(N = 46)	(N = 15)	
**Preoperative circulating blood examination**				
Platelets (n/mL)	57	239.0 (200.0–277.0)	227.5 (193.2–239.2)	*p* = 0.10
WBC (n/mL)	57	6.7 (5.1–7.7)	6.3 (5.2–7.4)	*p* = 0.72
Lymphocytes %	55	30.5 (24.9–33.8)	22.6 (24.9–27.1)	*p* = 0.01
Neutrophils %	55	60.1 (50.1–62.6)	66.3 (61.1–71.1)	*p* = 0.01
Monocites %	54	7.9 (6.8–10.0)	8.6 (6.1–10.4)	*p* = 0.93
CPR (mg/L)	42	2.9 (2.6–2.9)	3.1 (2.6–5.0)	*p* = 0.34
NLR %	55	1.9 (1.4–2.5)	3.1 (2.3–3.7)	*p* < 0.01
**Day 1 after surgery circulating blood examination**				
Platelets (n/mL)	60	216.5 (190.9–252.3)	218.5 (174.6–238.6)	*p* = 0.38
WBC (n/mL)	60	10.6 (9.3–13.5)	10.5 (8.9–14.0)	*p* = 0.73
Lymphocytes %	48	10.6 (8.4–16.5)	7.5 (5.6–10.0)	*p* = 0.06
Neutrophils %	46	78.4 (68.9–82.1)	85.4 (77.7–88.1)	*p* = 0.02
Monocites %	46	9.1 (7.8–10.0)	7.8 (5.7–8.9)	*p* = 0.13
**Day 30 after surgery circulating blood examination**				
Platelets (n/mL)	52	33.8 (18.0–48.2)	32.3 (20.3–64.0)	*p* = 0.75
WBC (n/mL)	25	6.9 (5.2–9.3)	6.4 (5.2–9.2)	*p* = 0.81
Lymphocytes %	25	26.0 (17.9–37.1)	28.0 (17.9–31.7)	*p* > 0.99
Neutrophils %	25	57.4 (43.6–67.1)	64.1 (53.4–70.2)	*p* = 0.31
Monocites %	25	6.4 (4.1–8.0)	6.1 (6.1–11.1)	*p* = 0.60
CPR (mg/L)	4	9.5 (1.9–32.8)	NA	NA
NLR %	25	2.3 (1.6–3.4)	2.3 (1.8–4.1)	*p* > 0.99

Abbreviations: WBC: White Blood Cells; CPR: C-Reactive Protein. NLR: Neutrophil-to-Lymphocyte ratio. N is the number of the sample. NA refers to Not Available. Comparisons were investigated with the Wilcoxon’s test.

**Table 5:** Univariate and Multivariate Cox Regression analysis including TIME with pathological variables available into the pathological report.

Variables	Status	n (%)	HR (Univariable)	HR (Multivariable)
TIME	Active	37 (74.0)	-	-
	Silent	13 (26.0)	4.84 (1.20–19.54, *p* = 0.027)	7.57 (1.49–38.57, *p* = 0.015)
STAS	No	27 (54.0)	-	-
	Yes	23 (46.0)	1.56 (0.39–6.32, *p* = 0.531)	1.88 (0.28–12.46, *p* = 0.511)
Resection margin	<10 mm	17 (34.0)	-	-
	≥10 mm	33 (66.0)	1.57 (0.32–7.55, *p* = 0.577)	4.08 (0.51–32.81, *p* = 0.186)
Histotype	Lepidic	48 (96.0)	-	-
	Others	2 (4.0)	2.10 (0.26–16.95, *p* = 0.487)	0.82 (0.06–10.70, *p* = 0.879)
Necrosis	<10%	31 (62.0)	-	-
	≥10%	19 (38.0)	1.42 (0.38–5.30, *p* = 0.601)	1.04 (0.18–6.13, *p* = 0.967)
Fibrosis	<10%	25 (50.0)	-	-
	≥10%	25 (50.0)	1.26 (0.34–4.73, *p* = 0.729)	1.71 (0.36–8.05, *p* = 0.499)
VI	No	24 (48.0)	-	-
	Yes	26 (52.0)	4.82 (0.60–38.78, *p* = 0.139)	3.71 (0.31–44.96, *p* = 0.304)

HR: Hazard Ratio, TIME: Tumor Inflammatory Microenvironment, STAS: Spread Through Air Spaces, VI: Vascular Invasion. Data are presented as Hazard Ratio (95% Confidence Interval, *p*-value).

## Data Availability

The original contributions presented in this study are included in the article/Supplementary Material. Further inquiries can be directed to the corresponding author.

## Supplementary Materials

The following supporting information can be downloaded at: https://www.mdpi.com/article/10.3390/jcm13247597/s1, Figure S1. First day post-operative values of peripheral circulating white blood cells and platelets; Figure S2. Linear regression for OS at postoperative oncological peripheral blood evaluation.
