# Supplementary material for: Peripheral Circulating Blood Cells Deviation Based on Tumor Inflammatory Microenvironment Activity in Resected Upstaged Lung Adenocarcinomas
Source: J Clin Med. 2024 Dec 13;13(24):7597. doi: 10.3390/jcm13247597 (PMC11676159; doi:10.3390/jcm13247597)
Supplement: Supplementary file 1 [file jcm-13-07597-s001.zip › jcm-3357397-supplementary.pdf]

Journal of
Clinical Medicine

MDPI

Supplementary Materials:

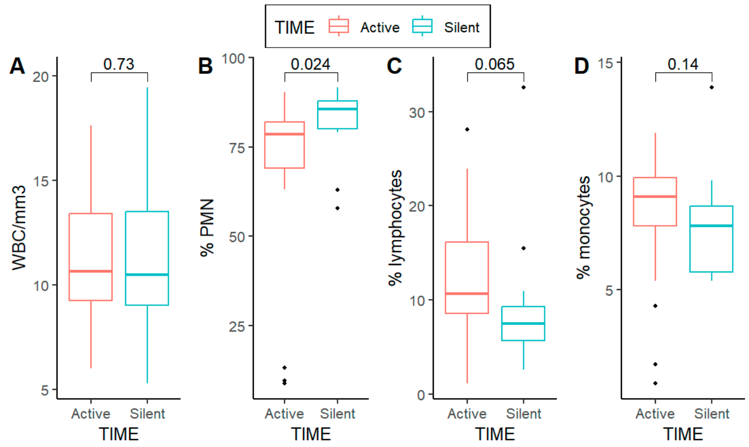

Figure S1. First day post-operative values of peripheral circulating white blood cells and platelets.

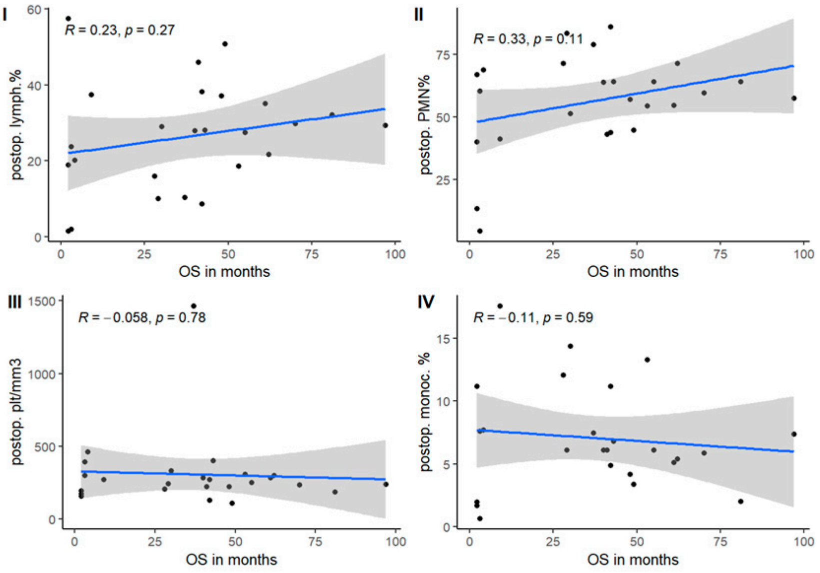

Figure S2. Linear regression for OS at postoperative oncological peripheral blood evaluation.
